# Do orthopaedic shoes improve local dynamic stability of gait? An observational study in patients with chronic foot and ankle injuries

**DOI:** 10.1186/1471-2474-14-94

**Published:** 2013-03-14

**Authors:** Philippe Terrier, François Luthi, Olivier Dériaz

**Affiliations:** 1 IRR, Institut de Recherche en Réadaptation, Sion, Switzerland; 2 Clinique romande de réadaptation SuvaCare, Sion, Switzerland; 3 Dpt de l'appareil locomoteur, FBM/CHUV, UNIL, Lausanne, Switzerland; 4 Clinique romande de réadaptation, Collaborateur scientifique / Service de recherche, Avenue Grand-Champsec 90, Sion, CH, 1951, Switzerland

**Keywords:** Foot orthoses, Footwear outcome, Gait variability, Ankle fractures, Lyapunov exponents

## Abstract

**Background:**

Complex foot and ankle fractures, such as calcaneum fractures or Lisfranc dislocations, are often associated with a poor outcome, especially in terms of gait capacity. Indeed, degenerative changes often lead to chronic pain and chronic functional limitations. Prescription footwear represents an important therapeutic tool during the rehabilitation process. Local Dynamic Stability (LDS) is the ability of locomotor system to maintain continuous walking by accommodating small perturbations that occur naturally during walking. Because it reflects the degree of control over the gait, LDS has been advocated as a relevant indicator for evaluating different conditions and pathologies. The aim of this study was to analyze changes in LDS induced by orthopaedic shoes in patients with persistent foot and ankle injuries. We hypothesised that footwear adaptation might help patients to improve gait control, which could lead to higher LDS:

**Methods:**

Twenty-five middle-aged inpatients (5 females, 20 males) participated in the study. They were treated for chronic post-traumatic disabilities following ankle and/or foot fractures in a Swiss rehabilitation clinic. During their stay, included inpatients received orthopaedic shoes with custom-made orthoses (insoles). They performed two 30s walking trials with standard shoes and two 30s trials with orthopaedic shoes. A triaxial motion sensor recorded 3D accelerations at the lower back level. LDS was assessed by computing divergence exponents in the acceleration signals (maximal Lyapunov exponents). Pain was evaluated with Visual Analogue Scale (VAS). LDS and pain differences between the trials with standard shoes and the trials with orthopaedic shoes were assessed.

**Results:**

Orthopaedic shoes significantly improved LDS in the three axes (medio-lateral: 10% relative change, paired t-test p < 0.001; vertical: 9%, p = 0.03; antero-posterior: 7%, p = 0.04). A significant decrease in pain level (VAS score -29%) was observed.

**Conclusions:**

Footwear adaptation led to pain relief and to improved foot & ankle proprioception. It is likely that that enhancement allows patients to better control foot placement. As a result, higher dynamic stability has been observed. LDS seems therefore a valuable index that could be used in early evaluation of footwear outcome in clinical settings.

## Background

Ankle and hind-foot fracture is one of the most common lower limb fractures [1]. Typical incidence rates (UK) are 15 per 10’000 person-years for ankle fractures and 12 per 10’000 for foot fractures [2]. After foot and ankle injury, the rehabilitation process usually includes the treatment of abnormal range of motion and the restoration of muscle strength [1,3]. Sometimes, especially in case of complex fractures such as calcaneum fractures or Lisfranc dislocations, patients may continue to have chronic functional limitations due to anatomical and biomechanical impairments, often associated with degenerative changes [4]. The use of various types of foot orthotics may help to improve the patient’s ambulatory status [5]. In order to provide better foot control, shoe modifications are used to accommodate and compensate for deformities, to eliminate painful motion, and to improve the rocking of the foot (rocking soles). The final therapeutic goal is to reach a better quality of life by enhancing ambulation and encouraging participation in daily living activities. For instance, it has been observed that orthotic insoles [6] or prescription footwear [7] can diminish pain and improve gait symmetry, and hence to enhance ambulation.

While prescription footwear is widely used in rehabilitation settings, there is still a need to better assess the footwear outcome on locomotor capabilities and gait quality. A non-linear approach has been proposed to characterize the Local Dynamic Stability (LDS) in continuous walking. It can be quantified by the system maximal Lyapunov exponent, which is the maximal average rate of divergence in pseudo-periodic processes [8]. LDS evaluates the ability of locomotor system to maintain continuous motion by accommodating infinitesimally small perturbations that occur naturally during walking [9]. This includes external perturbations induced by small variations in the walking surface, as well as internal perturbations resulting from the natural noise present in the neuromuscular system [9]. LDS has been advocated as a relevant indicator for evaluating traumatic injuries and orthopaedic surgery outcome [10,11]. Chang et al. [12] observed that walking on a compliant surface can be distinguished from overground walking by the maximal Lyapunov exponent. It has been reported that the absence of tactile sensation at plantar level (desensitization by ice) induced lower LDS [13]. Therefore, it could be hypothesized that changes in sensitive and proprioceptive feedbacks from the foot, and hence footwear modification, may influence LDS.

The objective of the present study was to analyze gait stability (LDS) in patients with chronic impairments after foot and ankle injuries. The effect of orthopaedic shoes was tested as compared to standard shoes. LDS was computed from accelerations signals measured at trunk level during short walking trials. The hypothesis was that pain relief, and improved foot control provided by prescription footwear would allow patients to better control his/her gait, and hence enhance dynamic stability.

## Methods

### Participants

Twenty-five patients (5 females, 20 males) were included in the study. Female patients were under-represented at the clinic, where the study took place. The participants’ characteristics were (mean (SD)): age 48 yr (16), body mass 82 kg (15), and height 173 cm (7). They signed an informed consent and the study was approved by the local ethics committee (Commission Cantonale Valaisanne d'Ethique Médicale, Sion, Switzerland).

They were hospitalized during an average of four weeks in a rehabilitation clinic (Clinique romande de réadaptation SUVAcare, Sion, Switzerland) and treated for post-traumatic impairments and disabilities following ankle and/or foot fractures. The aim of the therapeutic program was to take care of patients with a multidisciplinary approach in order to improve quality of life, functional status and chance of returning to work. During the hospitalization, inpatients follow active individual and group therapies (exercises, joint mobilization, etc.) every day for a mean time of 2.5 hours. The primary inclusion criteria were ankle and/or foot fractures with persistent impairments and footwear adaptation for treating pain and gait asymmetry. Included patients should be able to sustain 2 min of continuous walking. Those patients performed short walking tests for assessing gait regularity and symmetry [7]. From a database of accelerometric data obtained in 47 patients between 2005 and 2011, 22 patients have been discarded because of incomplete data, very low walking speed or stopping during walking tests. We finally selected 25 patients that maintained a minimal cadence of 85 steps/minute in four trials (two with standard shoes and two with orthopaedic shoes), in order to obtain 4x40 steps of steady gait (see below). The cadence ranged from 85steps/min to 124steps/min.

The diagnoses were malleolar fractures (N = 18, from which 4 were bi-malleolar and 2 tri-malleolar), mid- and hind-foot fractures (N = 11, 4 calcaneum fractures, 2 talus fractures, 1 cuboid fracture, 1 Chopart’s dislocation, 3 Lisfranc dislocations), tibial pilon and fibular fractures (N = 7), and metatarsal fractures (N = 4). The time after accident was about eight months. All patients took analgesic medication or non steroidal anti-inflammatory drugs (NSAIDs) and/or weak opioid without change during the testing period.

The AOFAS (American Orthopaedic Foot and Ankle Society) Hindfoot or Midfoot Questionnaires [14] were used to assess the patients’ status. The AOFAS score was calculated at the beginning and at the end of the hospitalization by taking into account the functional level of foot and ankle and the location of the fractures. The purpose of the AOFAS score was to assess the degree of severity of disabilities in order to better characterize the included patients.

Three patients were equipped with stabilizing shoes (ankle boots), one with open shoes and 21 with standard orthopaedic shoes (low shoes). A description of the prescription footwear is provided in a previous article [7]. Both feet were prescribed, taking into account to the specific demands of unilateral and bilateral injuries: in case of unilateral injury, shoe of the healthy side was adapted to provide symmetry with the injured side. The orthopaedic shoes were customizable models (such as Etonic®/Xelero® or Kunzli®), which were tailored to the needs of each patient. All shoes were equipped with rocker soles [15], with an height of 40 mm to 45 mm. By comparison, rocking height of a normal shoe typically lies between 10 mm and 15 mm. The rocker axis was aligned to the metatarso-phalangial joint, if any with a more distal/proximal position according to the corrective requirements. Several shoes parameters were adapted to individual disabilities, such as soles rigidity (further enhancement of foot rocking), lateral reinforcements (lateral stabilization of hindfoot), shoes volume (pain relief in case of oedema) or heel height (angle correction to the ankle joint). Footwear was completed with thermoforming custom-made orthoses (insoles): the aim was to stabilize the foot, to distribute the loads more evenly, to relief pressure over painful areas, and to correct foot malalignments. In case of unilateral injury, the healthy side also receive an orthoses for symmetry purpose. In addition, for patients exhibiting painful heel, orthoses contained a heel shock absorber.

### Experimental procedure

The motion sensor (*Physilog system, BioAGM, Switzerland*,) was a triaxial accelerometer connected to a data logger recording trunk accelerations in antero-posterior (AP), vertical (V), and medio-lateral (ML) directions. The sensor was attached to the lower back with an elastic belt at the L3-L4 level. The dimensions of the logger were 130 mm × 68 mm × 30 mm and its weight was 285 g. The accelerometers are piezoresistive sensors coupled with amplifiers (±5 g, 500 mV/g). In 15 patients, we used a newer version of the Physilog device with similar characteristics, except a smaller logger size (94 mm × 58 mm × 25 mm). The logger was worn on the side of the body by means of a shoulder strap. Signals were sampled at 200 Hz. After the experiment, data were downloaded to a computer. Subsequent data analyses were performed with MATLAB (*Mathworks, USA*).

The trials took place between one and three days after introduction of the orthotic treatment. Consequently, patients had only a short period of familiarization with the new shoes. During the control trials, participants wore their own usual shoes (Standard Shoes, SS), that they used most of the time at the clinic before the introduction of orthopaedic shoes. The patients walked at self selected speed along a 70-m corridor during 30s with the portable accelerometer recording their trunk acceleration. Four measurements were made: two control trials and two trials with orthopaedic shoes (OS). The sequence was: (1) 30-s walking. (2) 3-s break (U-turn). (3) 30-s walking. (4) changing shoes (1–2 min). (5) 30-s walking. (6) 3-s break (U-turn) and (7) 30-s walking. The order was balanced among participants: 14 subjects performed the test with SS at the beginning, and 11 in the reverse order (OS first).

Twenty-four patients reported levels of pain related to walking using a Visual Analogue Scale (VAS [16]) after the two first walking trials and at the end of the test in order to differentiate the effect induced by prescription footwear. The VAS was graduated from 0 to 100 mm (0 = no pain, 100 = extreme). We experienced one missing value due to organizational issues.

From the 30 sec raw acceleration measurement, the average Step Frequency (SF) was computed from Fast Fourier Transform (FFT). After discarding two seconds at the beginning of each test, a duration corresponding to 40 steps (20 strides) was kept for subsequent analysis. Hence, each acceleration set had a different time length (and hence variable sample length, ranging from 3866 to 6096) but an identical step number (40).

### Data analysis

The method for quantifying the local dynamical stability of gait by using largest Lyapunov exponent has been extensively described in a previous study [17]. It examines structural characteristics of a time series that is embedded in an appropriately constructed state space defined by time delay and embedding dimension. The state space was reconstructed according to the Takens’ theorem, as classically applied in gait dynamics studies [9]. Embedding dimension and time delay were assessed by using respectively Global False Nearest Neighbors (GFNN) analysis and Average Mutual Information (AMI) function. A time delay was defined for each signal according to AMI analysis. A constant dimension of 6 was set for all directions, according to the average GFNN results. As defined by the Rosenstein’s algorithm [8], the mean exponential rate of divergence of initially nearby points in the reconstructed space served to compute logarithmic divergence diagrams [18]. The maximum finite-time Lyapunov exponents (λ* [8]) were estimated from the slopes of linear fits in those diagrams. Strictly speaking, due to the non-linearity of divergence curves [17], multiple slopes could be defined. Hence, no true single maximum Lyapunov exponent exists. The slopes (exponents) quantify local divergence at different time scale, and should not be interpreted as a “classical” maximal Lyapunov exponent [17]. Time was normalized by average stride time for each subject and each condition. The slope over one stride was used (short term stability). The results of the two trials in each condition were averaged.

### Statistical analysis

**Figure 1:**
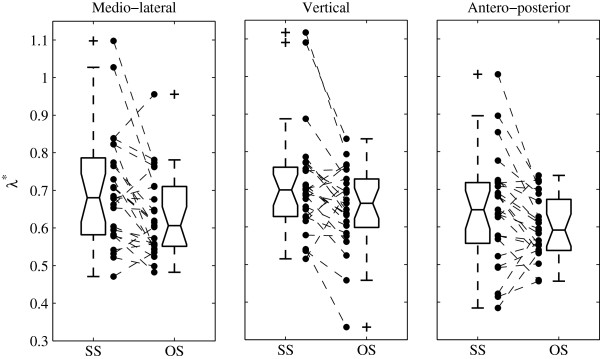
**Individual changes of dynamic stability induced by orthopaedic shoes.** Individual results of local dynamic stability (Maximal finite time Lyapunov exponents (λ^*^), i.e. rate of divergence) are presented. Discontinuous lines join the individual results of control trials with Standard Shoes (SS) and Orthopaedic Shoes (OS) trials (N = 25). Boxplots show the quartiles and the median. Columns represent the 3 axes of trunk acceleration, as measured by the triaxial accelerometer.

**Figure 2:**
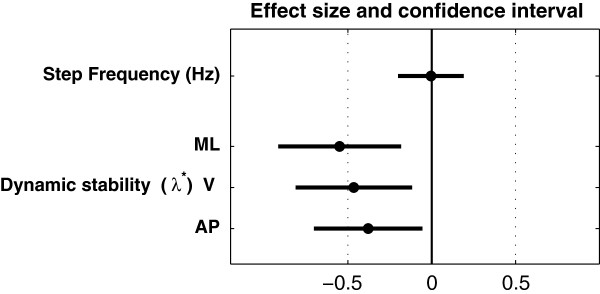
**Effect size: changes induced by orthopaedic shoes.** Black circles are the standardized effect size (Hedges’s g). Horizontal lines are the 95% confidence intervals, computed as ±1.96 times the Standard Error. The arbitrary limit of 0.5 (vertical dotted line) corresponds to a medium effect as defined by Cohen. Dynamic stability is defined as the logarithmic rate of divergence (finite time Lyapunov exponent, λ^*^). ML, V and AP stand for Medio-Lateral, Vertical and Antero-Posterior accelerations, i.e. the 3 axes of trunk acceleration as measured by the triaxial accelerometer.

The range of the individual data is presented as scatter- and box-plots (Figure 1). Mean and Standard Deviation (SD) were computed (Table 1). Ninety-five percent Confidence Intervals (CI) were calculated as ±1.96 times the Standard Error of the Mean (SEM). The effect size of orthopaedic shoes was expressed in both relative (Table 1) and standardized (mean difference divided by SD, Figure 2) terms. The standardized effect size was the Hedges’s g, which is a modified version of the Cohen’s d for inferential measure [19]. Paired t-tests between orthopaedic shoes and standard shoes were performed, and the p-values are shown in the last column of Table 1. The precision of the effect sizes was estimated with CI (±1.96 times the asymptotic estimates of the standard error, Figure 2). The arbitrary limit of 0.5 was uses to delineate medium effect size, as defined by Cohen [19].

**Table 1:** Descriptive statistics

		**Standard shoes**			**Orthopaedic shoes**				
**N = 25**		**A. Mean**	**(SD)**	**Confidence interval**	**B. Mean**	**(SD)**	**Confidence interval**	**Relative change**	**T-test (p)**
Step frequency (Hz)		1.76	(0.18)	1.69–1.82	1.76	(0.17)	1.70–1.82	0.0%	0.51
	ML	0.70	(0.15)	0.64–0.76	0.63	(0.10)	0.59–0.67	-10%	0.00
Dynamic stability (λ*)	V	0.71	(0.14)	0.65–0.76	0.65	(0.11)	0.60–0.69	-9%	0.03
	AP	0.64	(0.15)	0.58–0.70	0.60	(0.08)	0.56–0.63	-7%	0.04

Values are expressed as mean (N = 25), Standard Deviation (SD) and 95% Confidence Interval (mean ±1.96 times the Standard Error of the Mean). Relative change is the average percentage of change induced by orthopaedic shoes (i.e. column B minus column A divided by column A). The t-test column shows the p values of paired t-tests. Step frequency is the number of steps per second (walking cadence). Dynamic stability is defined as the logarithmic rate of divergence in the acceleration signals (finite time Lyapunov exponent, λ^*^). ML, V and AP stand for respectively Medio-Lateral, Vertical and Antero-posterior accelerations, i.e. the 3 axes measured by the triaxial accelerometer.

## Results

Figure 1 shows the individual results and the extent of the data (box plots). A majority of patients (N = 25) exhibited a higher stability (lower λ^*^ = > higher LDS) with orthopaedic shoes: 19 for medio-lateral axis, 18 for vertical axis and 17 for antero-posterior axis.

Table 1 presents the descriptive statistics of the sample, and the results of the paired t-tests. It confirms that LDS was significantly improved by orthopaedic shoes along the three axes. The relative effect ranged from 7% change (antero-posteriorl) to 10% (medio-lateral). Conversely, the cadence (Step Frequency) was not different (-0.0%) in the trials with orthopaedic shoes, and the result is clearly not significant.

Pain, as evaluated by VAS, was lower with orthopaedic shoes. The average score (max = 100 mm, N = 24) changed from 45 mm(19) (mean(SD)) to 32 mm(16), indicating a significant change (paired t-test, p = 0.002). The relative decrease was -29%, and the effect size was -0.76. At the beginning of hospitalization, the average AOFAS score (N = 25) was 57(14) and was improved (effect of the overall rehabilitation process) by 26% to 72(16) at the end of the stay.

Figure 2 presents the effect size of wearing orthopaedic shoes. It supports the hypothesis that footwear improves local dynamic stability of walking. A medium effect size is most likely for medio-lateral axis (d = -0.55). The two other axes exhibited a smaller effect size (vertical, d = -0.46; antero-posterior, d = -0.38). The absence of cadence change is confirmed.

## Discussion

In the present study, we performed gait analysis of 25 patients with persistent impairments after foot and ankle injuries during hospitalization in a rehabilitation clinic. The experimental procedure consisted of four short walking trials, wearing a lightweight 3D-accelerometer. We compare walking with and without orthopaedic shoes. The cadence remained unchanged between conditions. On the other hand, the results indicated that footwear improved local dynamic stability (LSD) of the gait (defined by using Lyapunov exponents). A significant effect was observed along the three axes, but it was more pronounced in the medio-lateral direction. Moreover, a substantial pain reduction was also observed.

In a previous study, we observed that orthopaedic shoes improved gait symmetry and reduced pain in patients with unilateral ankle injury [7]. The gait symmetry was defined as the difference between healthy side and injured side in the accelerometric signal. In the present study, it was possible to include patients with unilateral and bilateral impairments, because local dynamic stability is computed over consecutive gait cycles. Therefore, this parameter may be used in a broader spectrum of pathological gait than symmetry assessment. The study was conducted in a clinical setting: therefore there are strong evidences that the method we used is readily applicable by practitioners: collecting acceleration data during short walking tests is a simple and rapid procedure (5 min). Unlike most kinetics/kinematics measures, the short walking tests can be performed by the regular medical staff directly on site. However, LDS calculation still requires custom software and advanced computer skills.

Non-linear indexes of gait variability have attracted growing interest as clinically valid indicators for the follow-up of various pathologies [20,21]. LDS has already been shown relevant in the evaluation of various conditions such as in patients with peripheral arterial disease [22], in unilateral transtibial amputees [23], in knee osteoarthritis [10], or in Anterior Cruciate Ligament (ACL) rupture [11]. LDS has been also used to study the effect of aging and fall risks in elderly [24-26]. The relative change in LDS induced by orthopaedic shoes lay between 7% and 10%. In order to evaluate the relevance of this change, we compared it to similar results in the literature: Manor et al. [13] reported that desensitization of the foot soles (induced by ice-exposure) led to LDS decrease by -40%. Sinitksi et al. [27], by applying visual and mechanical perturbations to walking individuals, found that LDS was reduced by about -25%. By impairing balance control with application of randomly varying Galvanic Vestibular Stimulation (GVS), van Schooten et al. [28] reported an effect on LDS of -11%. We reported that treadmill walking significantly increased LDS as compared to over-ground walking by +9% [17]. The effect of aging on LDS, defined as relative difference between young and older adults, has been found to be about -70% [29] or -40% [24]. On the other hand, some studies failed to report significant effects on LDS: for instance, no LDS change was reported after a single bout of resistance exercise (effect of fatigue: 0%). Walking without arm swing led to a non-significant slightly higher LDS (+3%). The values of the present study are on the lower end of the range of reported effects; in absolute value they are similar to the stabilizing effect of treadmill or to the destabilizing effect of GVS: although orthopaedic shoes did not stabilize gait in a large extent, it should be noted that most of the experimental studies that we examined report the effect of destabilizing interventions [13,27,28]. Conversely, we evidenced a non-invasive therapeutic intervention that immediately improve LDS, what, as far as we know, has no equivalency in the literature..

Although the study was conducted in a single centre, included patients came from all over the west part of Switzerland. Apart the gender imbalance, they were therefore well representative of the chronic foot&ankle injured population in Switzerland. Although there was a substantial diversity among the diagnoses (midfoot, hindfoot and ankle injuries), included patients were treated by a homogenous and constant team of specialists (physical medicine practitioners and orthotists), which ensured that the orthopaedic shoes had a constant design during the study. The AOFAS score is widely used to assess the degree of impairment in this type of patient, and can serve as a basis to generalize the results to other comparable populations. The average score at hospitalization time (57) is low as compared to other studies [30,31], what reflects important disabilities and substantial pain that justified hospitalization. The significantly higher score at the end of stay (76) was the result of the multiple therapeutic interventions, including prescription footwear. Deeper analysis of AOFAS results goes beyond the scope of the present study and would require further investigations.

Speed was not assessed during the trials. Therefore, under the null hypothesis that footwear adaptation had no effect, we have to exclude the possibility that a significant speed difference occurred “by chance” between conditions, that would have induce a change in LDS. It is well established that SF is tightly related to walking speed [32,33]. Because we observed that cadence was not significantly modified between conditions, it is likely that speed remained also unchanged. However, if a significant effect of orthopaedic shoes is hypothesized, it is possible that a step length change occurred, which induced increased speed without cadence modification. Effectively, some results in the literature support the fact that foot orthoses might improve step length more than cadence [34]. As a result, it is possible that the LDS increase was concomitant to step length improvement. It should be noted that the relationship between LDS and speed is still a matter of debate [35]. The results seem to show that there is little LDS modification around preferred walking speed [35] or that LDS decrease with walking speed (higher λ_s_[36,37]): consequently, it is very unlikely that the reported LDS increase was exclusively induced by higher speed.

Due to short acclimation time with new orthopaedic shoes (1-3 days), we did not evaluate the long-term beneficial effects of prescription footwear. Further follow-up studies are therefore needed. However, in a clinical context, it is crucial to evaluate the treatment effects rapidly, in order to adapt further interventions [7]. Consequently, LDS is likely a responsive index that would be pertinent for the early evaluation of footwear outcome.

Classically, non-linear analyses of the gait variability (such as Lyapunov exponent, or Detrended Fluctuation Analysis), have been performed over long continuous walking trials [38-40]. It has been shown that short term LDS could be assessed with a good reliability with a trial of at least 2 minute [41] or 150 consecutive strides [42]. It has been suggested that type II errors could be frequent with short walking samples. However, increasing the number of subjects [42] and multiplying the number of measured walking episodes [43] has been proposed to attenuate this effect. Consequently, two episodes of 20 strides and 25 patients have been used in the present study. Other studies successfully used LDS in short walking trials of 30 strides [22,24,37], 16 m walking distance [26], or 8 strides [44]. Although it is likely that long duration trials could provide a higher precision, the results show that LDS was responsive to footwear change despite the low number of analyzed strides.

Treadmill walking has been shown to influence walking variability and stability [39,40], questioning its use for gait evaluation. In addition, many patients cannot walk for a long period because of fatigue and pain. It has been shown that LDS was modified while turning as compared to walking in a straight line [45]. A straight path should therefore be used to evaluate LDS in order to avoid potential bias induced by turns. Based on these considerations, the experimental design of the present study was to submit patients to four 30s trials along a straight corridor. It was a balance between a) the need to maximize the length of the trials in order to increase the precision on LDS, b) the necessity to avoid long and painful walking sessions, and c) the space limitation of the building (70 m corridor).

We observed that footwear effect was stronger for the medio-lateral axis (d = 0.55), and barely significant for antero-posterior direction (p = 0.04). Other studies also highlighted discrepancies between the different axes [26]. With a comparable method (trunk acceleration), Chang et al. [12] observed significant differences in LDS between normal and compliant surfaces for medio-lateral and vertical axes, but not for antero-posterior axis, i.e. the same trend as in our results. Furthermore, it has been observed that, under dual tasking conditions (Stroop test), LDS was responsive along the three axes, with a lower sensitivity in AP direction [42]. It is unclear whether these differences could have a biomechanical significance, however, it is worth noting that theoretical studies emphasize the role of lateral stability in falls avoidance [46].

Concerning pain, we measured a significant (p < 0.01) and practically relevant positive effect of the orthopaedic shoes (medium effect size: -0.76). Recent recommendations have stated that pain reductions ≥30% —a threshold close to our results (28%)— appear to reflect a least moderate clinically important differences [16].

What are the underlying mechanisms that could explain the observed improvement in local dynamic stability? Ankle injuries, and more specifically lateral ankle sprains, are often associated with chronic ankle instability. Both mechanical (joint laxity) and functional (proprioceptive disorders) instabilities have been described [47]. Several studies related ankle instability to postural control deficit [48] and altered kinematics and kinetics [49]. It has been shown that biomechanical changes induced by ankle instability are due to altered neuromuscular control [49], even at supraspinal level [50]. The role of foot orthoses in the treatment of ankle instability has been emphasized [51]. It has been proposed that improvement of tactile sensation could be a mechanism by which orthoses are beneficial to postural control [51]. Furthermore, ice-induced plantar desensitization led to decreased LDS (approximately -40%), what bring evidence of relationships between foot sensitive feedbacks and gait stability. The functional impairments and disabilities of subjects included in the present study (AOFAS score: 57) goes far beyond the after-effects of simple lateral ankle sprain, such as functional instability. Most of the studied patients suffered from important foot and/or ankle pain while walking. Many exhibited mechanical restrictions at the ankle joint and/or at the subtalar joint. Moreover, it is obvious that severe foot and ankle fractures impair the proprioceptive and sensitive feedbacks at the foot and ankle level. In addition, it can be hypothesized that pain interferes with the motor control both at peripheral and supraspinal level (avoidance reflex). The results of the present study and previous results demonstrated that orthopaedic shoes reduce pain [7]. Taking together those considerations and evidences in the literature about ankle instability, we hypothesize that the improvement in local dynamic stability was mainly due to pain reduction and/or enhanced proprioceptive/sensitive feedbacks provided by the orthopaedic shoes. The improvement in tactile sensations may have allowed the patients to better control their injured foot and hence to reduce noise in the motor control, that resulted in a better dynamic stability of gait. This hypothesis is also reinforced by the fact that most patients received low orthopedic shoes, which did not provide a strong mechanical support to the ankle: therefore, the explanation of a pure mechanical stabilization should be discarded. Because each patient received specific prescription footwear with multiple adjusted parameters, it is of course still unclear what is the exact cause of the observed results and which combination of adaptations would be more efficient for gait stabilization. However, the advice could be, if enhanced gait stability is part of the therapeutic goals, that more stress should be put on pain relief and foot comfort than on pure mechanical foot stabilization. The benefits of enhanced foot control may be an increased confidence in walking, with a concomitant reduction of stumbling and hence a lower risk of re-injury, but that remains to be investigated.

## Conclusions

Foot orthotics and prescription footwear are designed in order to accommodate and compensate for deformities, and to eliminate painful motion [5]. However, the outcome of the intervention in terms of gait recovery is variable among patients. As a result, the assessment of footwear outcome needs rapid, objective and reproducible method, which evaluates the gait more thoroughly than the simple observation [7]. Accordingly, the present study brings further insights into the non-linear analysis of short walk test in clinical settings. Among other parameters, the local dynamic stability could help the clinicians to improve the intervention (for instance, modifying the footwear). However, further studies are needed to translate the results of the present study in a workable diagnostic tool. Practically, dedicated software for end-users should be made available to facilitate the calculation of LDS. Furthermore, test-retest trials should be conducted to better understand the intra-individual variability of LDS. Finally, the effect of footwear for specific type of injury (i.e. maleolar, hind-foot, metatarsal fractures…) should be established in a larger population.

## Abbreviations

LDS: Local Dynamic Stability; SD: Standard deviation; AOFAS: American Orthopaedic Foot and Ankle Society; AP: Antero-Posterior; V: Vertical; ML: Medio-Lateral; SS: Standard Shoes; OS: Orthopaedic shoes; VAS: Visual Analogue Scale; CI: Confidence Intervals.

## Competing interests

The authors declare that they have no competing interests.

## Authors’ contributions

PT, OD and FL designed the study. PT collected and analyzed the data and wrote the manuscript. FL contributed to the analysis (AOFAS score) and to the revision of the manuscript. OD supervised the study and participated to the revision of the manuscript. All authors read and approved the final manuscript.

## Pre-publication history

The pre-publication history for this paper can be accessed here:

http://www.biomedcentral.com/1471-2474/14/94/prepub
